# Military Personals

**Published:** 1918-04

**Authors:** 


### MILITARY PERSONALS

Capt. A. L. Benedict has been transferred from Base Hospital at Camp Sheridan, Ala., to Officers Training Camp, Ft. Oglethorpe, Ga.

To Camp Travis, Fort Sam Houston, Texas, Lieut. Frank E. Fox, Fulton.

To Fort Riley, Lieut. Samuel T. Nicholson, Jr., Clifton Springs.

To Macon, Ga., Lieut. John A. Morrissey, Newark.

To New York City, Capts. Harry Weed, Buffalo; Charles W. Hoyt, Rochester; Lieuts. F. P. Schenkelberger, Collins; Elias C. Fischbein, Sonyea; Wm. L. Grogan, Rome.

To their homes and honorably discharged on account of physical disability, Capts. Francis W. McGuire, Buffalo; James E. Holden, Collins; James H. Irish, Syracuse.

To Camp Custer, Battle Creek, Lieut. James L. Horndorf, Rochester.

To Camp Dix, Wrightstown, N. J., Major Martin B. Tinker, Ithaca; Capt. Roland C. Meisenbach, Buffalo.

To Fort McHenry, Md., Lieut. Maxwell C. Montgomery, Rome.

To Fort Oglethorpe, Capt. Charles H. Erway; Lieuts. Earl W. Thema, Buffalo; Harry E. Wheelock, Jamestown.

To report to Surg.-General of the Army, Lieut. Earl H. Lormor, Buffalo.

To Rochester, Capt. Clayton K. Haskell, Rochester; Lieuts. Raymond J. Brown, Eric S. Green, Rochester.

To Army Medical School, Wash., Lieuts. Chas. G. Irish, Lancaster; Wm. M. Edmonds, Tonawanda.

To Washington, Lieut. Martin F. Nolan, Tonawanda.

To Allentown, Pa., Lieut. Elmer E. Owen, Batavia.

To Camp Devens, Ayer, Mass., Lieut. Adelbert C. Abbott, Syracuse.

To Camp Dix, Wrightstown, Lieut. John A. Metzen, Buffalo.

To Camp Wadsworth, Spartansburg, S. C., Lieuts. Jos. A. Nowicki, Buffalo; Herbert L. Kallet, Syracuse.

To New York City, Capts. Edward T. Wentworth, Rochester; Samuel Stewart, Syracuse; Lieuts. Arthur C. Smith, Elmira; Ira W. Livermore, Gowanda; Joseph L. Wozniak, Buffalo.

To Philadelphia, Pa., Lieut. Arwin H. MacFarland, Rome.

To Pittsburgh, Pa., Lieut. Bert G. Voorhees, Elmira.

To Rockefeller Institute, Capt. Chas. I. Hincher, Rochester.

